# Energy research with neutrons (ErwiN) and installation of a fast neutron powder diffraction option at the MLZ, Germany^1^

**DOI:** 10.1107/S1600576718004223

**Published:** 2018-04-13

**Authors:** Michael Heere, Martin J. Mühlbauer, Alexander Schökel, Michael Knapp, Helmut Ehrenberg, Anatoliy Senyshyn

**Affiliations:** a Institute for Applied Materials (IAM), Karlsruhe Institute of Technology (KIT), Hermann-von-Helmholtz-Platz 1, 76344 Eggenstein-Leopoldshafen, Germany; b Heinz Maier-Leibnitz Zentrum (MLZ), Technische Universität München, Lichtenbergstrasse 1, 85748, Garching bei München, Germany

**Keywords:** neutron powder diffraction, *operando* techniques, *in situ* studies, spatially resolved characterization, energy storage

## Abstract

The need for rapid data collection and studies of small sample volumes in the range of cubic millimetres are the main driving forces for the concept of a new high-throughput monochromatic diffraction instrument at the Heinz Maier-Leibnitz Zentrum (MLZ), Germany. A large region of reciprocal space will be accessed by a detector with sufficient dynamic range and microsecond time resolution, while allowing for a variety of complementary sample environments. The medium-resolution neutron powder diffraction option for ‘energy research with neutrons’ (ErwiN) at the high-flux FRM II neutron source at the MLZ is foreseen to meet future demand. ErwiN will address studies of energy-related systems and materials with respect to their structure and uniformity by means of bulk and spatially resolved neutron powder diffraction. A set of experimental options will be implemented, enabling time-resolved studies, rapid parametric measurements as a function of external parameters and studies of small samples using an adapted radial collimator. The proposed powder diffraction option ErwiN will bridge the gap in functionality between the high-resolution powder diffractometer SPODI and the time-of-flight diffractometers POWTEX and SAPHiR at the MLZ.

## Abstract 2

A fast and medium-resolution neutron powder diffraction option for ‘energy research with neutrons’ (ErwiN) at the high-flux FRM II neutron source at the Heinz Maier-Leibnitz Zentrum (MLZ) is described.

## 1. Introduction   

Energy storage is one of the great challenges for present and future generations. As humanity tries to reduce the use of CO_2_-intensive energy production pathways and adopt renewable ones, it is necessary to store renewably generated energy for times of great demand, as a direct consequence of the inherently intermittent means by which such energy is produced, *i.e.* sun and wind. In Germany, power production has changed during the past decade, especially after the meltdown in Fukushima, Japan. Germany decided to undertake an *Energiewende*, which translates as ‘a drastic change in energy production’. With that in mind, renewable energy production has been increased, and the current need for energy storage systems inspired the project ErwiN (energy research with neutrons), with opportunities to investigate energy-related materials *in situ* and *operando* with thermal neutrons. Among other energy-storage technologies, the focus will primarily be on Li-ion batteries. Moreover, other fields of research could benefit from a fast neutron powder diffraction instrument. For example, *in situ* hydrogenation measurements on materials suitable for concentrated solar power (Sheppard *et al.*, 2014 ▸; Humphries *et al.*, 2016 ▸) or hydrogen storage (Heere, GharibDoust *et al.*, 2016 ▸; Heere, Sørby *et al.*, 2016 ▸; Gharib­Doust *et al.*, 2016 ▸; Heere, GharibDoust, Brighi *et al.*, 2017 ▸; Dolci *et al.*, 2010 ▸; Heere, GharibDoust, Sørby *et al.*, 2017 ▸; Frommen *et al.*, 2015 ▸) could help researchers to understand reaction pathways. Furthermore, *operando* multi-parameter studies could be undertaken to follow structural changes in magnetic and electrical fields (Ehrenberg *et al.*, 2012 ▸; Gilmore *et al.*, 2018 ▸) or in ferroelectric materials (Hinterstein *et al.*, 2015 ▸), or to carry out simultaneous electrochemical and powder diffraction measurements on batteries (Senyshyn *et al.*, 2013 ▸). With the second detector, spatially resolved diffraction studies can be carried out, *e.g.* to determine the lithium distribution inside Li-ion cells (Senyshyn *et al.*, 2014 ▸, 2015 ▸; Mühlbauer *et al.*, 2017 ▸).

ErwiN itself will be a new and fast neutron powder diffraction option at the FRM II neutron source in Munich, and it will also update and complement the current RESI instrument at beamport SR8b – a single-crystal diffractometer using thermal neutrons (Pedersen *et al.*, 2006 ▸). In this work, we present the future experimental setup of ErwiN, which will share the beamport and primary neutron optics with the RESI diffractometer. The commissioning of ErwiN started in 2017 and the instrument is expected to be operational by mid-2019.

## 2. Proposed experimental setup   

**Figure 1:**
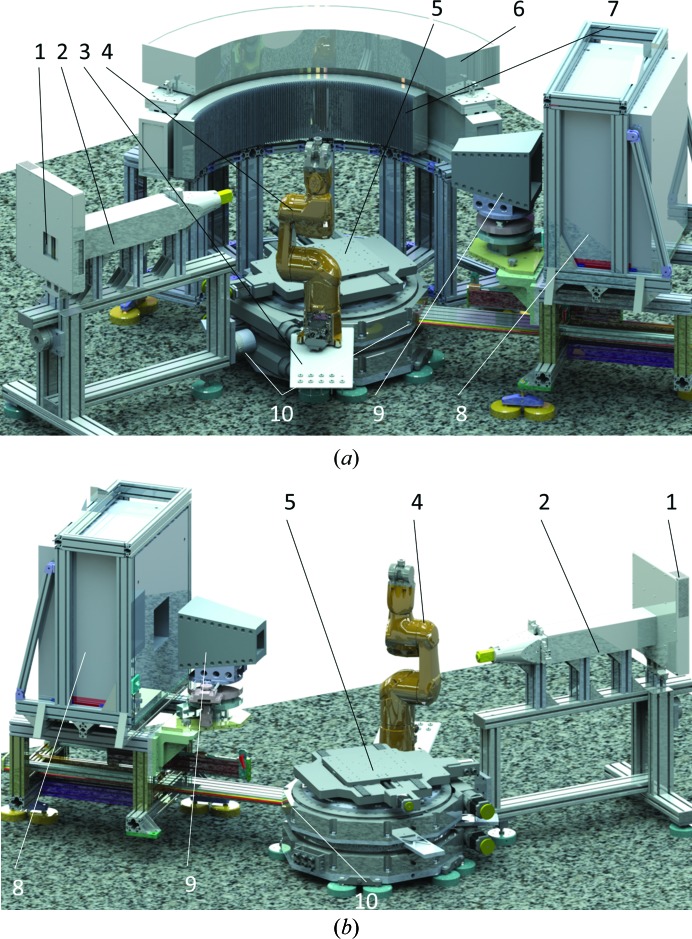
(*a*) A sketch of ErwiN with two detectors. The components are numbered as follows: (1) monochromatic beam exit, (2) secondary flight path, (3) stock of vanadium sample containers for robotic sample changer, (4) six-axis robotic arm, (5) sample table (*x*, *y*, *z*, ω, θ_1_, θ_2_, θ_3_), (6) large curved two-dimensional multidetector (*r*_i_ = 800 mm, *h* = 200 mm, 2θ coverage 135°), (7) radial oscillating collimator, (8) 30° two-dimensional neutron detector for spatially resolved powder diffraction, (9) radial oscillating collimator and (10) fast mechanical remounting plugs. (*b*) A rear view of the setup for the spatially resolved powder diffraction option (with the large curved two-dimensional multidetector removed).

The project ErwiN and its proposed experimental setup are inspired by two world-class high-throughput powder diffractometers operating with monochromatic neutrons, namely D20 at the Institut Laue–Langevin (ILL), Grenoble, France (Hansen *et al.*, 2008 ▸), and WOMBAT at the Australian Centre for Neutron Scattering (ACNS), ANSTO, Sydney, Australia (Studer *et al.*, 2006 ▸). The principle of the ErwiN setup is presented in Fig. 1 ▸, where Fig. 1 ▸(*a*) shows the complete scheme while Fig. 1 ▸(*b*) elucidates the rear view and, therefore, the view on the ‘narrow’ 30° curved two-dimensional detector. Fig. 1 ▸ includes the monochromatic beam exit (labelled 1) [including the pyrolytic graphite (PG)/Ge/Cu monochromators]. The secondary flight path (labelled 2) comprises a fast shutter, a neutron monitor, a horizontal Soller collimator, a PG filter and a specially designed slit system. The Soller collimator and PG filter are pneumatically driven, while adjustment of the slit system will be performed by a combination of linear stages (not shown in Fig. 1 ▸). The sample table is designed for handling heavy sample environments up to 1000 kg, such as vertical superconducting magnets and tensile rigs. The installation of the Eulerian cradle of the current RESI setup will allow for texture measurements or offer the functionality of a four-circle diffractometer.

The stock of vanadium sample containers (labelled 3 in Fig. 1 ▸ *a*) with up to 100 samples will be handled by a six-axis robot (labelled 4; Stäubli TX2-60 CS9) for fully automated sample changing with a standard gripping system by Co. Schunk. The robotic arm will serve as a sample stage with *x*, *y*, *z*, ω, χ translation capabilities for small samples (nominally 3.5 kg, maximum 9 kg, 30 µm positional accuracy) for bulk and spatially resolved neutron powder diffraction.

The sample table (labelled 5) consists of three goniometer tables (θ_1_, θ_2_, θ_3_). Additionally, the sample table can be moved ±125 mm in the *x*, *y* and *z* directions, with an additional rotational ω axis installed on top (not shown in Fig. 1 ▸).

**Figure 2:**
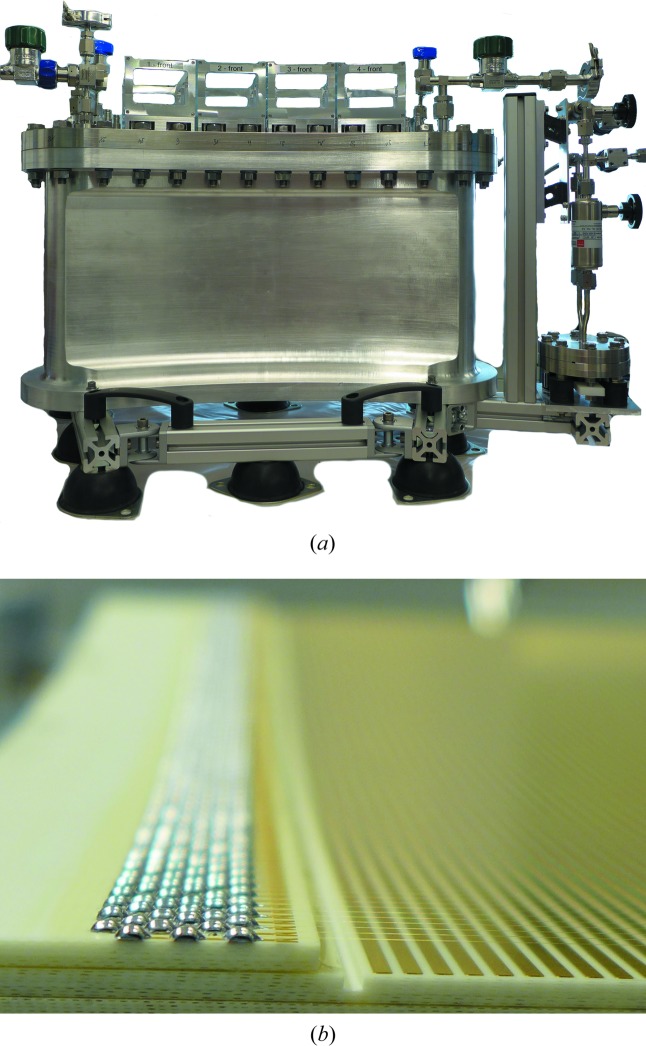
(*a*) The prototype of the 30° MWPC two-dimensional neutron detector (2 × 15°) for spatially resolved powder diffraction was built as a collaboration between the FRM II detector group, the Paul Scherrer Institute (PSI), Switzerland, and the Institut Laue–Langevin (ILL), France. In contrast with the prototype, the larger curved two-dimensional multidetector will be equipped with nine of the 15° modules. A pumping system is attached to filter the ^3^He + CF_4_ gas mixture. (*b*) The cathode and anode layers are arranged in sandwich mode. From the top: cathode (50 µm wires), anode (15 µm wires) and cathode (PCB strips). The curvature (*r*_i_) is 0.8 m.

The large curved two-dimensional multidetector [multiwire proportional chamber (MWPC), labelled 6] will have a 2θ coverage ≥135° (based on curved segments of 15° 2θ each), with an effective sample-to-detector distance of 0.80 m (inner radius of curvature *r*_i_ = 0.8 m) and a vertical sensitive area of 0.20 m (corresponding to ν ≃ 15°). The unwanted scattering signal of the sample environment will be suppressed by a radial oscillating collimator (labelled 7). The powder option is designed to deliver diffraction patterns on a two-dimensional detector from an ∼1 cm^3^ sample volume in only a few minutes and from a *ca* 10–30 mg sample in 5–15 h. The large curved two-dimensional multiwire chamber is considered to be the main detection system for ErwiN. Based on the initial design of Brookhaven National Laboratory, USA (Langan *et al.*, 2004 ▸), installed on the Protein Crystallography Station at LANL (now at ORNL, Tennessee, USA), and on the WOMBAT diffractometer (Studer *et al.*, 2006 ▸) at ACNS, the new modification of the detector will be built by the detector group at FRM II. A two-segment (2 × 15° in 2θ) prototype (Fig. 2 ▸) has already been developed and built and was tested in late 2017. The physical detector resolution is expected to be better than 1.5 mm, with a maximum count rate of 200 000 counts s^−1^ per segment (a rate limit of 1.8 MHz for the whole detector) and event-mode detection with a nanosecond timestamp.

It is planned that the prototype of the curved two-dimensional multidetector will become operational at the end of 2018. It will be used as a ‘narrow’ 30° two-dimensional neutron detector (labelled 8) for spatially resolved powder diffraction and will remain at the instrument alongside the larger curved two-dimensional multidetector. Fast mechanical remounting plugs (dowel pins) are installed to enable fast exchange of the sample table and detectors (labelled 10).

Fig. 1 ▸(*b*) presents the setup for the spatially resolved powder diffraction option at ErwiN, while Fig. 2 ▸ shows the 30° two-dimensional neutron detector as it is currently being tested at the TREFF neutron reflectometer at the MLZ. Experiments with minimum gauge volumes in the region of 1 mm^3^ are foreseen, which require a sophisticated radial oscillating collimator (9) with a field of view of ∅ < 1 mm (∅ < 0.5 mm as an option). Data will be collected by the 30° two-dimensional neutron ^3^He detector, and the effect of 1.5 mm detector resolution on the instrument resolution can be compensated by the sample-to-detector distance of 0.8 m, which will result in an angular (2θ × ν) detector coverage of 30° × 15°.

**Figure 3:**
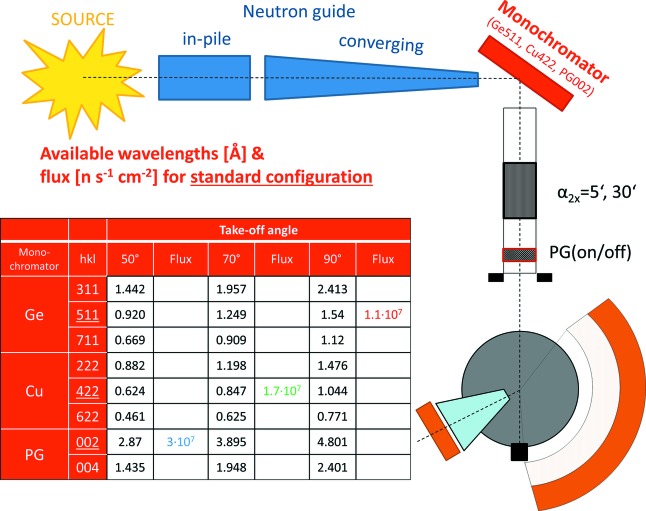
The available wavelengths, flux and layout/scheme of the ErwiN option at the MLZ.

The proposed setup for ErwiN will benefit from the rather short neutron guide and therefore a location close to the reactor vessel, with a distance of only 8.4 m from the reactor polygon to the monochromator. This combination of a bright neutron source and a short distance provides a high neutron flux (see the table in Fig. 3 ▸) and achieves a neutron beam cross section from 70 × 40 mm (h × v) at the reactor polygon down to 60 × 30 mm (h × v) in front of the monochromator. The level of background is minimized by heavy concrete shielding (1.2 m thick). At the moment, the single-crystal option RESI employs discrete exits, equivalent to three fixed take-off angles. The first option is a 90° take-off angle with two alternative monochromator options, the 511 reflection from a focused Ge monochromator and the 422 reflection from a Cu monochromator, with mosaic spreads of 20 and 25′, respectively. Two other options with take-off angles of 50 and 70° are available and result in configurations with increased neutron flux. Although the configuration at 50° take-off will only be available by switching the detector position to the defocused diffractometer side, it is considered a reasonable instrument configuration as it reaches the highest flux at the sample position.

The powder diffraction option ErwiN will share beamport SR8b with the single-crystal diffractometer RESI, which will preserve all its present functions. The available beam time will be allocated between ErwiN and RESI upon specific requests. For a quick change between the two instrument layouts, all parts of ErwiN are designed to be mobile, namely the secondary flight path, the sample goniometer, the curved two-dimensional detector and the setup for spatially resolved diffraction. They will be attached to each other *via* mechanical dowel pins, enabling easy remounting with a reproducibility better than 100 µm.

The instrument will be operated by the *NICOS* control system, a common software solution controlling instruments at the MLZ (https://forge.frm2.tum.de/wiki/). Event-mode recording and high rates of data collection place high demands on fast instrument control, communication protocols and data storage. The required computing capabilities will be specified in more detail after finalizing the results of testing the first detector prototype in late 2017.

## 3. Simulations   

**Figure 4:**
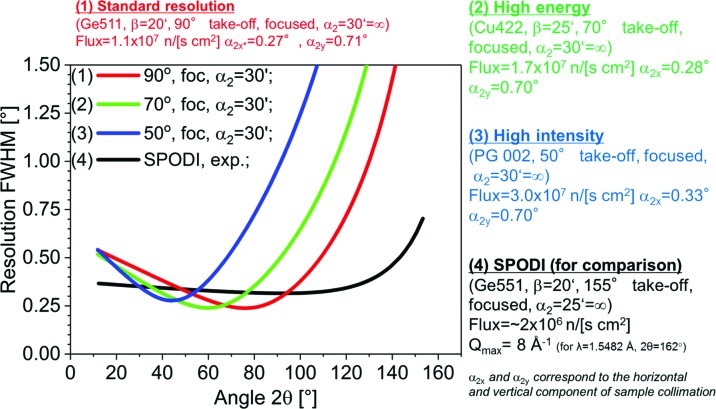
Expected neutron flux and peak resolution as determined from ray-tracing simulations using *McStas* (Lefmann & Nielsen, 1999 ▸) compared with the SPODI resolution function.

Ray-tracing Monte Carlo simulations (*McStas*; Lefmann & Nielsen, 1999 ▸) have been performed for the proposed powder option using the existing primary neutron optics and monochromators of SR8b (Fig. 4 ▸). In the present monochromator configuration (Ge511 and Cu422) only short wavelengths can be accessed at low take-off angles, considerably limiting the resolution of the setup in the high-intensity option. Therefore, a vertically focusing PG monochromator with 30′ mosaicity (see the table of available wavelengths in Fig. 3 ▸) is proposed to be installed as a third monochromator in the RESI casemate. With this suite of monochromators, a broad range of wavelengths (0.46–4.8 Å) can be selected from the thermal spectrum at three take-off angles of beamport SR8b. Neutron fluxes, divergence profiles and resolution curves in the form of real two-dimensional diffraction patterns were calculated for three configurations, namely (1) standard resolution, (2) high energy and (3) high intensity (Lefmann & Nielsen, 1999 ▸). The results are summarized in Figs. 3 ▸ and 4 ▸. The available *Q* ranges for all options are given in Table 1 ▸. The experimental resolution function of the high-resolution diffractometer SPODI (Hoelzel *et al.*, 2012 ▸) is shown for comparison in Fig. 4 ▸.

Configurations (1) and (3) are foreseen for the standard operation of ErwiN, where neutron fluxes differ by a factor of three and the minimum of the resolution curve is shifted depending on the take-off angle (compare Fig. 3 ▸). The neutron flux at ErwiN is predicted to be five to ten times higher than that at SPODI in its standard configuration (155° take-off angle). Along with the advantages of the stationary detector (no need to reposition to collect a complete diffraction pattern; compare this with SPODI, which makes either 20 or 40 resolution steps in order to complete a diffraction pattern in the angular range 10–160° in 2θ), this may give an overall gain of up to 100 in data collection efficiency, thus reducing the required exposure times from hours to minutes in the standard-resolution configuration. The ErwiN option will thus open new fields of kinetic time-resolved, multi-parametric and spatially resolved *operando* neutron diffraction experiments at the MLZ. By its functionality, not only is ErwiN complementary to the existing high-resolution powder diffractometer SPODI but, with its monochromatic beam, it is also complementary to the time-of-flight medium-resolution diffractometers POWTEX and SAPHiR, which are under construction.

## 4. Conclusions and future outlook   

The ‘fast’ powder diffraction option (ErwiN) at beamport SR8b at the MLZ is an attractive instrumental solution for the neutron science user community and complements the existing and future instrumental pool at the MLZ. Furthermore, the location of ErwiN in the ‘diffractometers’ corner’ of the experimental hall has a number of advantages regarding the transfer of sample environment, equipment and experimental infrastructure. The development of a medium-resolution powder diffraction instrument using a large two-dimensional detector option will boost internal and external research activities equally. This will also help to consolidate new concepts and to prove them.

The thermal neutron beam at SR8b is ideal for hosting the high-efficiency powder diffraction option ErwiN. This is underlined by the above-described beam conditions, optional take-off angles close to the direct beam and the availability of sufficient space for all experimental setups.

## Figures and Tables

**Table 1:** The available *Q* ranges (Å^−1^) for the wavelengths given in Fig. 3 ▸ *Q*_max_ was calculated for 2θ = 145°.

		Take-off angle		
Monochromator	*hkl*	50° *Q*_max_	70° *Q*_max_	90° *Q*_max_
Ge	311	8.31	6.12	4.97
	511	13.03	9.60	7.78†
	711	17.91	13.18	10.70
Cu	222	13.59	10.00	8.12
	422	19.21	14.15‡	11.48
	622	26.00	19.18	15.54
PG	002	4.18§	3.08	2.50
	004	8.35	6.15	4.99

† Corresponding to curve (1) (red) in Fig. 4 ▸.

‡ Corresponding to curve (2) (green) in Fig. 4 ▸.

§ Corresponding to curve (3) (blue) in Fig. 4 ▸.
